# Consuming an unprocessed diet reduces energy intake: a post-hoc analysis of a randomized controlled trial reveals a role for human nutritional intelligence

**DOI:** 10.1016/j.ajcnut.2025.101183

**Published:** 2025-12-29

**Authors:** Jeffrey M Brunstrom, Mark Schatzker, Peter J Rogers, Amber B Courville, Kevin D Hall, Annika N Flynn

**Affiliations:** 1 Nutrition and Behaviour Unit, School of Psychology and Neuroscience, University of Bristol, Bristol, United Kingdom; 2 National Institute for Health and Care Research (NIHR) Bristol Biomedical Research Centre: Nutrition Theme, University of Bristol, University Hospitals Bristol Education & Research Centre, Bristol, United Kingdom; 3 Modern Diet and Physiology Research Centre, McGill University, Montreal, QC, Canada; 4 National Institute of Diabetes and Digestive and Kidney Diseases, Bethesda, MD, United States

**Keywords:** ultraprocessing, NOVA, ultraprocessed foods, energy intake, micronutrient deleveraging, carbohydrate, fat, food choice, energy balance

## Abstract

**Background:**

In 2019, Hall et al. reported a randomized clinical trial showing that an ultraprocessed diet increases energy intake by ∼500 kcal/d compared with an unprocessed diet.

**Objective:**

This post-hoc analysis assessed whether participants selected meal components with specific nutritional characteristics and how this affected energy intake.

**Methods:**

Twenty weight-stable adults received an ad libitum ultraprocessed or unprocessed diet for 2 wk, followed by the alternate diet. ANOVA and *t* tests assessed diet effects; a linear mixed model assessed predictors of meal size.

**Results:**

With the unprocessed diet, participants selected components with a less-equal blend of energy from carbohydrate and fat [“blend index” difference; lunch = 0.22 (95% CI: 0.19, 0.26), *P*< 0.0001, *d* = 0.76; dinner = 0.24 (95% CI: 0.19, 0.28), *P*< 0.0001, *d* = 0.71]. These components formed meals that had a lower blend index (less balanced) than ultraprocessed meals [lunch, *F*(1, 19) = 18.49, *P* < 0.0004, partial *η*^*2*^ = 0.493; dinner, *F*(1, 19) = 24.85, *P* < 0.0001, partial *η*^*2*^ = 0.57]. With the unprocessed diet, participants preferentially chose low-energy-dense components (<1.0 kcal/g, mostly fruits and vegetables), creating meals lower in energy (unprocessed = 719.4 ± 11.6 kcal compared with ultraprocessed = 829.5 ± 12.51 kcal), [*F*(1,19) = 14.9, *P* < 0.001, *η*^*2*^*G* = 0.0457], yet significantly larger (57%) by mass (unprocessed = 665.5 ± 10.74 g compared with ultraprocessed = 423.5 ± 8.03 g), [*F*(1,19) = 82.9, *P* < 0.001, *η*^*2*^*G* = 0.274]. Modeled together, low-energy-dense mass and blend index strongly predict observed energy intakes (r = 0.78, df = 1676, *P* < 0.001).

**Conclusions:**

Unprocessed meals may reduce energy intake because: *1*) they have a less balanced carbohydrate-fat blend; and *2*) they promote a form of nutritional intelligence whereby a compromise is struck between consuming calories and consuming micronutrients, which we refer to as “micronutrient deleveraging.”

This trial was registered at clinicaltrials.gov as NCT03407053.

## Introduction

In 2019, Hall et al. [1] ran a 28-day metabolic ward study with 20 weight-stable adults. Participants were randomly assigned to 2 wk of an ultraprocessed diet followed by 2 wk of an unprocessed diet, or vice versa. Though meals were matched for calories, energy density, macronutrients, sugar, sodium, and fiber, participants nevertheless ate an average of 508 kcal/d more on the ultraprocessed diet, gaining 0.9 kg, and lost 0.9 kg on the unprocessed diet. Concern about ultraprocessed foods has grown, yet little attention has been paid to an equally relevant question: why did Hall et al.’s [1] participants consume fewer calories with unprocessed meals?

**FIGURE 1:**
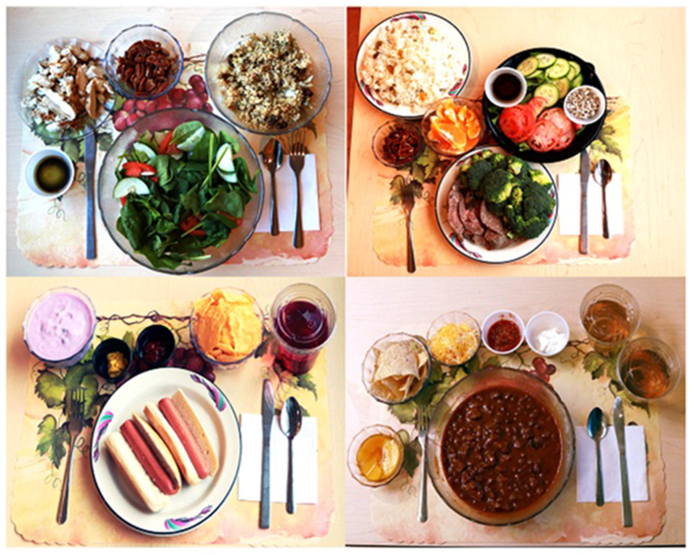
Examples of meals served to participants in a study reported by Hall et al. [1] (2019). Top row: unprocessed lunch (left) and dinner (right). Bottom row: ultraprocessed lunch (left) and dinner (right). These images were published in Cell Metabolism, 30(1), Hall et al. [1], Supplemental Material: Ultraprocessed diets cause excess calorie intake and weight gain: An inpatient randomized controlled trial of ad libitum food intake, 67-77, Copyright Elsevier (2019). Reproduced from reference [1] with permission.

A secondary analysis by Fazzino et al. [2] linked energy density, protein, eating rate, and “hyper-palatability” to energy intake. However, this type of “meal-level” analysis overlooks a key consideration: participants were instructed to eat “as much or as little as desired” and thus had the autonomy to choose amounts of individual meal “components,” each served in a large portion (Figure 1). In this post-hoc analysis, we therefore examine how the selection of components influenced energy intake.

We were guided by 2 recent discoveries related to “nutritional intelligence” [3]: the preferential selection of foods based on their nutritional composition. The first is the discovery that foods with a more equal blend of energy from fat and carbohydrate (e.g., croissants) are more rewarding than foods with equivalent calories from primarily fat (e.g., cheese) or carbohydrate (e.g., pretzels) [4], a finding that has been replicated in the United States and United Kingdom [5,6], and in nonhuman mammals [7]. The rewarding nature of “combination foods” has been attributed to the supra-additive combination of both fat and carbohydrate reward pathways [4,7]. Recently, Rogers et al. [6] argued that humans are adapted to select such foods because their higher “energy-to-satiety ratio” enables greater energy intake, and thus the selection of larger portions (kcals) (A.N. Flynn, P.J. Rogers, and J.M. Brunstrom, submitted).

Here, we sought to extend this to consider multicomponent meals. Specifically, we predicted that Hall et al.’s [1] participants selected meal components that produced a more balanced combined (meal-level) blend of carbohydrate and fat than the blend found in the individual components from which the meals were derived. Additionally, we explored 2 related questions: *1*) If participants combined sources of carbohydrate and fat to form their eaten meals, were meals with a more equal blend also higher in energy? and *2*) Did unprocessed meals have a less balanced blend of energy from carbohydrate and fat, potentially explaining why, when participants consumed them, they were lower in energy?

A second reason why an unprocessed diet might promote lower energy intake is that meal components may be selected for nutritional qualities other than calories. Early studies in chickens [8], pigs [9], and rats [10] showed that animals grow as well or better when provided free access to a cafeteria diet compared with formula feed. Building on this, Rozin [11] and Provenza [12,13] showed how species adjust their dietary choices to alleviate specific micronutrient deficiencies. Recently, we reported that humans might show a similar tendency, preferring specific pairs of fruit and vegetables that deliver a broad range of micronutrients [14]. Because fruits and vegetables are rich in micronutrients, yet low in energy density, we reasoned that consuming these components might lead to less calorific meals [15,16]. Thus, “micronutrient seeking” might be an additional factor contributing to lower energy intake from unprocessed meals in Hall et al.’s [1] study.

## Methods

As described above, the present paper reports further analyses of data collected in a previously published article by Hall et al. [1]. Full details of the methods are available in that article, including participant eligibility criteria and sample size calculations. A summary of those methods, together with the details of the present (new) data analysis, is presented below.

### Summary of Hall et al. 2019 [1]

Twenty (10 male and 10 female) weight-stable adults aged (mean ± SE) 31.2±1.6 y and BMI of 27 ± 1.5 kg/m^2^ were admitted as inpatients to the Metabolic Clinical Research Unit at the NIH Clinical Center, Bethesda, MD. Weekly menus were repeated twice, and so every participant received either an ultraprocessed or an unprocessed diet for 2 wk, followed by the alternate diet for 2 wk. Breakfast, lunch, dinner, and snacks were served daily, and foods and beverages were categorized according to the NOVA system [17]. Across the 2 wk, the unprocessed and ultraprocessed diets were matched for calories, sugar, fat, sodium, fiber, and macronutrients.

Participants ate alone and received portions equivalent to twice their estimated energy requirements. All foods were weighed to the nearest 0.1 g before and after consumption, and energy intake was calculated using ProNutra software (Viocare, Inc.). Details of participant characteristics, recruitment, exclusions, and adverse events can be found at https://clinicaltrials.gov/study/NCT03407053?tab=results. The Institutional Review Board of the National Institute of Diabetes & Digestive & Kidney Diseases approved the protocol. Data were collected between April 2018 and November 2018.

### Data analysis

Previously, Hall et al. [1] estimated the combined impact of food and beverages on absolute energy intake. Here, our primary concern was understanding how separate meal components (solid foods) combine to impact choice and energy intake. Thus, following Fazzino et al. [2], we looked at the 3 daily meals and excluded snacks and beverages from our analysis. In total, 1680 meals (20 participants × 14 d × 2 diets) were analyzed (Supplemental Figure 1).

Using additional data provided by Hall et al. [1], we grouped ingredient-level data into meal “components.” Here, we define a component as a food item that cannot be easily broken down into subcomponents when consumed. For instance, muesli and lasagna are formed from separate identifiable ingredients, but they would each be coded as a single meal component because their ingredients are premixed and consumed together. By contrast, peas and potatoes are single ingredients but are rarely combined; therefore, they will likely be categorized as separate components if they appear in the same dish. Respectively, unprocessed breakfasts, lunches, and dinners comprised on average 2.86, 4.14, and 5.0 components. Processed breakfasts, lunches, and dinners comprised 3.71, 3.64, and 4.14 components, respectively.

Following Rogers et al. [6], for each meal component, we calculated a transformed measure of food carbohydrate-to-fat “blend index” using the formula: ratio = 1 − ABS(2x − 1), where x = carbohydrate energy/ (carbohydrate energy + fat energy). A blend index can vary from 0 (contains carbohydrate but no fat energy, or contains fat but no carbohydrate energy) to 1 (contains equal amounts of carbohydrate and fat energy). We then did the same for whole meals consumed (again, nonbeverage energy only).

To quantify micronutrient intakes across the 2 diets, we assessed 15 vitamins and minerals (calcium, iron, magnesium, phosphorus, potassium, zinc, copper, vitamin C, thiamine, riboflavin, niacin, vitamin B6, folate, vitamin B12, and vitamin A). Following Brunstrom and Schatzker [14], for each meal component, we computed the extent to which it provided a recommended daily allowance (RDA, [18]). Separate values (%) were derived for the amount consumed and the amount remaining at the end of the meal.

Q–Q plots are included in the Supplementary Materials. They show predominantly at least a good fit of scores to a normal distribution [19]. In our analysis of how meal components vary in their “carbohydrate-to-fat blend index,” we observed a departure from normality. This can be problematic for sample sizes <20 [20]. However, the Central Limit Theorem ensures that hypothesis tests are robust against extreme skewness and kurtosis if sample sizes exceed 300 [21]. In this case, our sample exceeded 6000. Accordingly, no data transformations were applied, no data were missing, and no data points were excluded.

Weighted *t*-tests were used to compare how the composition (carbohydrate-to-fat blend index) of the eaten meal components differed across diets. Linear mixed-effect models were then used to determine how these variables impacted meal size. Models were fitted using the restricted maximum likelihood method with a random intercept for each participant. Continuous predictors were standardized.

Microsoft Excel and R statistical software (R Foundation for Statistical Computing) were used for data processing and analysis. Mixed-effects models were generated using the lme4 package. Unless indicated otherwise, results are presented as means ± SEMs.

## Results

### Participant characteristics

This data reanalysis was conducted in a cohort of 20 weight-stable adults (10 men and 10 women) with a mean (±SE) age of 31.2 ± 1.6 y and a mean BMI of 27.0 ± 1.5 kg/m^2^.

#### Did participants select an equal proportion (%) of each meal component?

**FIGURE 2:**
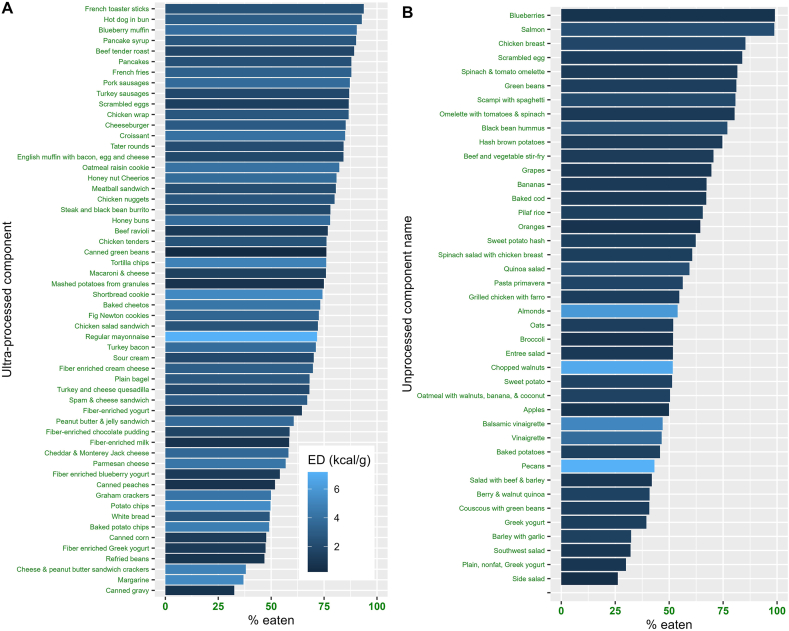
Proportion (%) of each meal component consumed. (**A**) Ultraprocessed (**B**) Unprocessed. Only components providing >100 kcal are shown. Components are color-coded according to their energy density (kcal/g). Lighter shades of blue indicate higher energy density. Note that the ultraprocessed fiber-enriched milk was served with breakfast cereal. ED, energy density.

In the first instance, we explored whether participants consumed the same amount of food, irrespective of the meal component provided. For each meal component, we computed the average energy consumed across meals and removed components that provided <100 kcal. This eliminated mostly condiments and other small food items; the remaining components still accounted for 89.3% of the total nonbeverage food energy served. Plotting ultraprocessed and unprocessed components separately, Figure 2 shows the proportions (%) consumed. For the ultraprocessed components (Figure 2A), this ranged from 93% for “French toaster sticks” to 33% for canned gravy. A similar range was observed in unprocessed foods (Figure 2B). Only 25% of the side salad was consumed, whereas 99% of the blueberries were eaten. Figure 2 also shows that when meal components are ordered by proportion (%) consumed, we see even distributions—with no distinct clusters of components eaten entirely, moderately, or hardly at all. Accordingly, we conclude that participants did not tend to consume a fixed proportion of every meal component. Instead, some components were preferentially selected and consumed over others. [Note that all components (including those < 100 kcal) were included in the analyses reported below].

#### Blend of energy from carbohydrate and fat in meal components and whole meals

Having established that meal components were selected and consumed in different amounts, we then looked at their blend of carbohydrate and fat and how individual components were selected and combined to influence the balance of carbohydrate-to-fat in whole meals.

**FIGURE 3:**
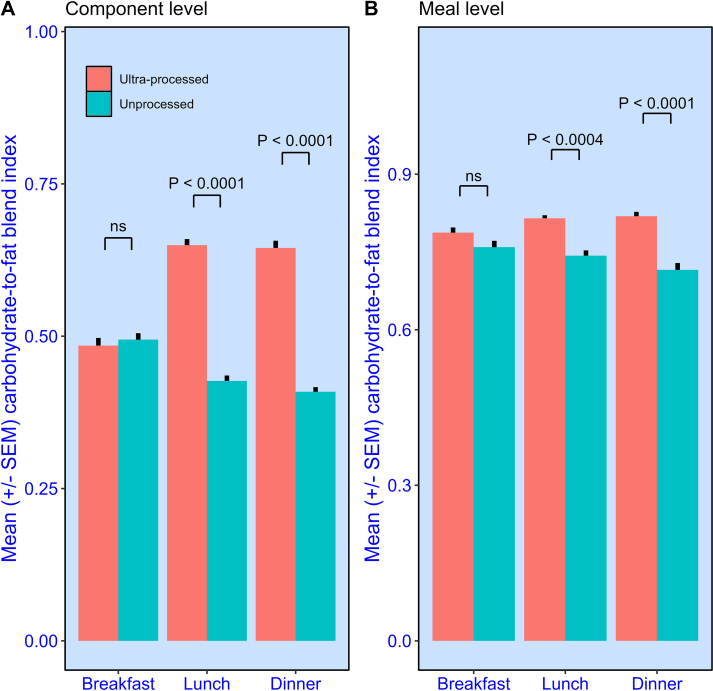
Mean (± SEM) transformed carbohydrate-to-fat blend index (0 = contains carbohydrate but no fat energy or contains fat but no carbohydrate energy; 1 = contains equal amounts of carbohydrate and fat energy). (**A**) Ultraprocessed and unprocessed meal components consumed. Means are weighted by the energy content of the components. (**B**) Ultraprocessed and unprocessed meals consumed. Separate values are provided for breakfast, lunch, and dinner. SEM, standard error of mean.

Figure 3A shows the carbohydrate-to-fat blend index of meal components eaten at breakfast, lunch, and dinner, and for the ultraprocessed and unprocessed diets, separately. Here, weighted means are shown, such that higher energy components contribute more to the overall average than lower energy components. At breakfast, the ultraprocessed and unprocessed components had a similar carbohydrate-to-fat blend index (weighted mean; ultraprocessed = 0.485 ± 0.016 and unprocessed = 0.494 ± 0.014) and did not differ significantly (weighted *t*(1000.8) = 0.462, *P* = 0.64, *d* = 0.028).

With lunch and dinner, however, we see a marked difference. In both cases, the ultraprocessed components had a significantly higher (lunch, weighted *t*(940.5) = 12.72, *P* < 0.0001, *d* = 0.76; dinner, weighted *t*(892.6) = 10.80, *P* < 0.0001, *d* = 0.71) carbohydrate-to-fat blend index (lunch = 0.649 ± 0.017 and dinner = 0.655 ± 0.017) than the unprocessed components (lunch = 0.427 ± 0.014 and dinner = 0.409 ± 0.017).

The same pattern was observed when components were combined to form meals (Figure 3B). Here, the carbohydrate-to-fat blend index of the ultraprocessed breakfasts was marginally higher than in the unprocessed diet (means; unprocessed = 0.759 ± 0.012 and ultraprocessed = 0.790 ± 0.009), although this difference failed to reach significance, *F*(1,19) = 2.07, *P* = 0.17, partial *η*^*2*^ = 0.098). In part, this might be because the ultraprocessed and unprocessed breakfast meal components tended to have a more similar blend index than other meals. By contrast, the ultraprocessed lunches and dinners had a significantly [lunch, *F*(1, 19) = 18.49, *P* < 0.0004, partial *η*^*2*^ = 0.493; dinner, *F*(1, 19) = 24.85, *P* < 0.0001, partial *η*^*2*^ = 0.57] higher carbohydrate-to-fat blend index (lunch = 0.8215 ± 0.006 and dinner = 0.8217 ± 0.007) than their unprocessed counterparts (lunch = 0.7430 ± 0.010 and dinner = 0.7154 ± 0.013).

Figure 3 also shows another clear difference between individual components and entire meals. Meals had a higher carbohydrate-to-fat blend index than the individual components from which they were formed. This can only happen if the participants combined components that were predominantly a source of fat with components that were predominantly a source of carbohydrate. A further possibility we wished to investigate is whether ultraprocessed meals had a higher overall carbohydrate-to-fat blend index because these meals were formed from components that already had a higher carbohydrate-to-fat blend index, so achieving a more balanced blend was “easier” for participants who received this diet.

**FIGURE 4:**
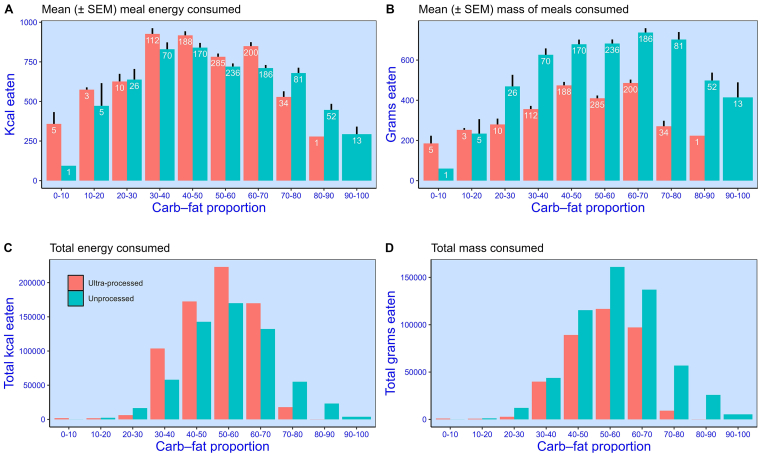
Meals binned (10% steps) according to their % energy from carbohydrate versus fat. (0 = contains carbohydrate but no fat energy, 50 = contains equal amounts of carbohydrate and fat energy, 100 = contains fat but no carbohydrate energy.) (**A**) Mean (± SEM) energy consumed (kcals). (**B**) Mean (± SEM) mass consumed (g). Mean (± SEM) daily nonbeverage amounts consumed by participants (*N* = 20) over the 2-wk diet. (**C**) Total energy (kcals) consumed. (**D**) Total mass (g) consumed. Separate values are provided for ultraprocessed and unprocessed meals. Values overlaid on bars (A and B) indicate the number of meals in each bin.

To understand the impact of specific amounts of carbohydrate and fat on energy intake, we compared the proportion of meal energy consumed from carbohydrate and fat across the 2 diets. In Figure 4A, meals are binned in 10% (0.1) steps according to whether the consumed amounts comprised almost exclusively fat (0%–10% carbohydrate) through to almost exclusively carbohydrate (90%–100% carbohydrate). Separate means are provided for each bin and diet, along with the number of meals in each bin.

Figure 4A shows that participants tended to choose and consume components that formed meals with a near 50:50 blend of energy from carbohydrate and fat. Moreover, as predicted, more energy (kcals) tended to be consumed for meals with a more equal blend. The fact that high-fat meals are lower in energy than more blended meals demonstrates that the higher energy intake associated with blending is driven by the combination of macronutrients and not the mere presence of fat (the more energy-dense macronutrient).

A comparison of unprocessed and ultraprocessed meals also revealed something unexpected. For consumed meals with a carbohydrate content in the range of 0% to 70%, more energy (kcals) was consumed for the ultraprocessed meals than the unprocessed meals. Although there are relatively few meals above this threshold (>70% carbohydrate), we see the converse tendency: more energy was consumed for the unprocessed meals.

Figure 4B shows the same binned data, this time for meals according to their mass (g). Here, the difference between unprocessed and ultraprocessed is more obvious. In almost all meals, a larger mass (g) of food was consumed when participants were given the unprocessed diet, and this difference is especially noticeable when meals comprised a higher proportion of carbohydrate-to-fat.

Figure 4A and Figure 4B capture how average meal size (energy and mass, respectively) varies with the proportion of consumed meal calories from carbohydrate and fat. However, because the frequency of meals varies across this range (they tend toward a balance of carbohydrate and fat), the absolute impact is challenging to visualize. Therefore, in Figure 4C and Figure 4D, we present the same data, this time for the total energy (C) and total mass (D) consumed across the 2 diets. The patterns in Figure 4A and Figure 4B are broadly replicated. Again, it is striking that participants consistently consumed a larger mass of food when given the unprocessed diet, and this is particularly the case in meals for which the consumed energy from carbohydrate exceeded the energy derived from fat.

### Meal size and energy density

**FIGURE 5:**
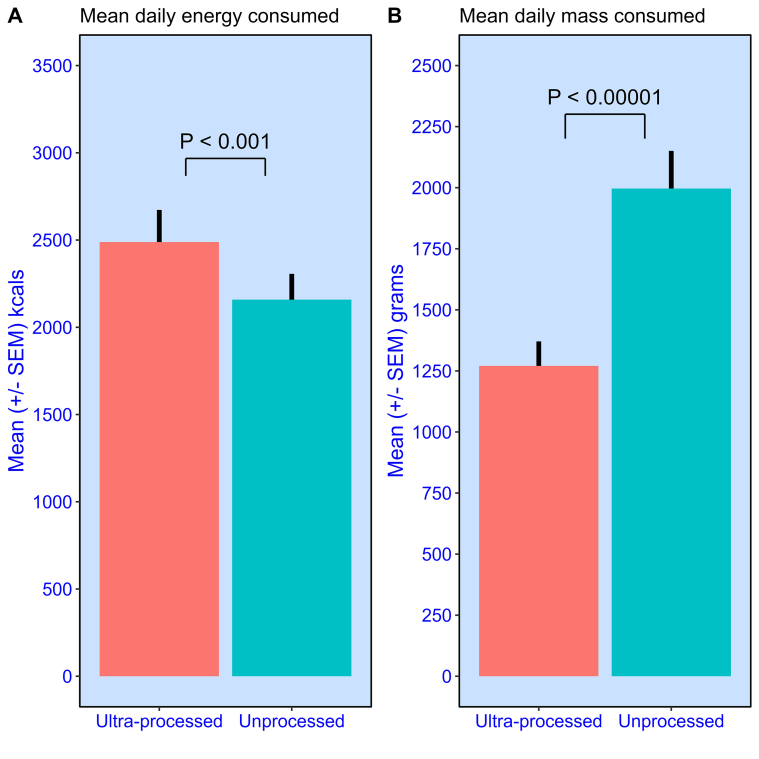
Mean (± SEM) daily nonbeverage amounts consumed by participants (*N* = 20) over the 2-wk diet. (**A**) energy (kcals). (**B**) mass (g). Separate values are provided for ultraprocessed and unprocessed diets.

Figure 5A shows the mean (± SEM) daily difference in nonbeverage energy consumed from meals across the 2 diets. Participants consumed 15.3% more calories in the ultraprocessed diet, equivalent to an additional 330 kcal per participant per day. However, the opposite is true with mass (Figure 5B). Here, the unprocessed diet encouraged participants to consume 57% more nonbeverage food, which amounts to an average additional 726 g per participant per day. Indeed, every participant (*N* = 20) exhibited this tendency. From this, we can conclude that participants consumed unprocessed meal components with lower energy density (ultraprocessed = 1.96 kcal/g; unprocessed = 1.08 kcal/g).

**FIGURE 6:**
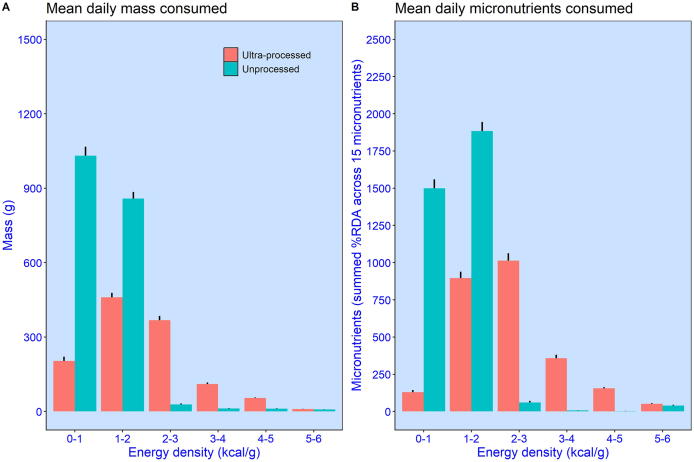
Mean (± SEM) daily nonbeverage amounts consumed by participants (*N* = 20) over the 2-wk diet. Values are binned (1 kcal/g steps) according to their energy density. (**A**) Mass consumed (kg). (**B**) Micronutrients (summed %RDA across 15 micronutrients). Separate values are provided for ultraprocessed and unprocessed meal components; RDA, recommended daily allowance.

For both diets, Figure 6A shows the mean (± SEM) daily mass of nonbeverage food consumed by participants (*N* = 20). Values are partitioned into separate energy density bins ranging from 0.0 to 1.0 kcal/g through to 8.0 to 9.0 kcal/g, in 1.0 kcal/g increments. Here, we see a clear difference. In the unprocessed diet, the greatest proportion of meal components was in the range of 0.0 kcal/g to 1.0 kcal/g, which comprises mainly fruits and vegetables. This amounts to 52% of the total mass of food consumed in the unprocessed diet. Thus, vegetable and fruit consumption probably played an important role in the tendency for these meals to have a relatively low-energy density. Moreover, because the unprocessed meals were significantly larger in mass, participants were not merely selecting the same physical amount of food across diets.

Why were low-energy-dense (<1.0 kcal/g) meal components consumed in such large portions (mass) in the unprocessed diet? To address this question, we investigated whether participants could have selected more energy-dense meal components, thereby raising meal-level energy densities to make them closer to the ultraprocessed diet. As Figure 2 indicates, many energy-dense meal components were not consumed in large amounts, and in many cases, lower energy-dense components were selected instead (e.g., blueberries, green beans, and bananas). We also note that, on average, in the unprocessed diet, participants left 370.1 kcals of >1.0 kcal/g meal components on their plate (compared with 217.8 kcals of <1.0 kcal/g). Had this been consumed, it might well have been sufficient to match the intake observed in the ultraprocessed condition. Thus, the tendency to select and consume very low-energy-dense components in the unprocessed diet was unlikely to be due to a lack of access to more energy-dense alternatives.

### Predicting meal-level energy intakes

Above, we report 2 key differences between the meal components consumed in the unprocessed and ultraprocessed diets. First, we see a relationship between meal size (kcals) and the tendency to select components that form meals with a higher carbohydrate-to-fat blend index (a more balanced blend of energy from carbohydrate and fat). In the case of the ultraprocessed diet, these meal-level carbohydrate-to-fat blend indexes were significantly higher than those associated with the unprocessed diet.

Second, we found that participants selected large portions (mass) of low-energy-dense meal components (<1.0 kcal/g) when they were offered the unprocessed diet. In turn, this helped to generate unprocessed meals that were significantly larger in mass, *F*(1,19) = 82.9, *P* < 0.00001, *η*^*2*^*G* = 0.274, yet significantly lower in energy, *F*(1,19) = 14.9, *P* < 0.001, *η*^*2*^*G* = 0.0457 (Figure 5A and 5B).

Isolating and quantifying the independent role of these 2 outcomes is challenging because selecting low-energy-dense components might limit meal size in 2 ways. First, it adds volume to the stomach, limiting the amount of more energy-rich food that can be comfortably consumed. Second, because fruits and vegetables comprise primarily carbohydrates, their preferential selection will tend to bias the meal-level carbohydrate-to-fat blend index, which, as we have seen, also predicts meal size. In response, we opted to estimate the impact of both factors in a single regression model. To generate a crude estimate of the tendency to select and consume low-energy-dense components, for each meal, we computed a “fruit and vegetable score” based on the fraction of energy that was derived from meal components with an energy density of <1.0 kcal/g.

**FIGURE 7:**
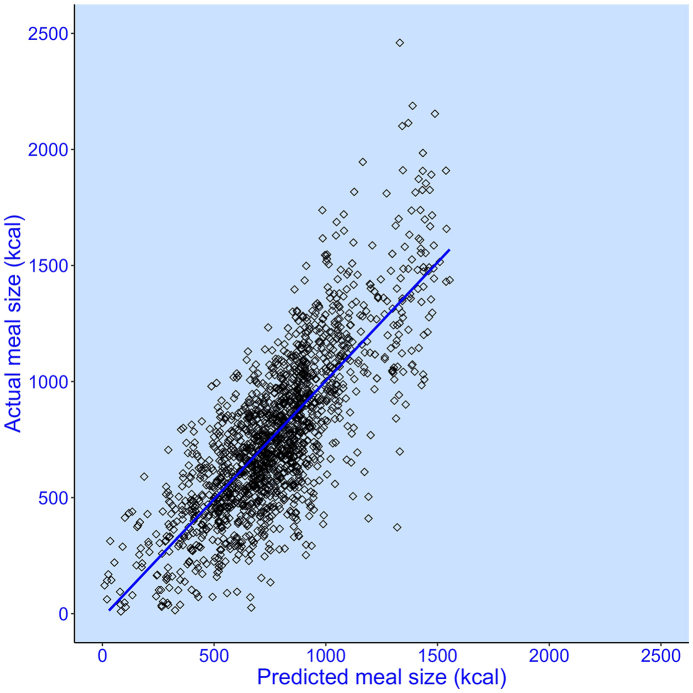
Modeled versus measured meal energy intake. A linear mixed-effects model included 3 fixed factors: *1*) carbohydrate-to-fat ratio, *2*) “fruit and veg score” (tendency to consume components with an energy density <1.0 kcal/g), and *3*) meal type (breakfast, lunch, dinner), to predict ad libitum energy intake. The model also included “participants” as a random factor with an independent intercept for each participant. Data show 1678 meals consumed by 20 inpatient adults exposed to an ultraprocessed and an unprocessed diet.

Following Fazzino et al. [2], along with meal type, we entered these variables into a mixed-effects model as predictors of the energy consumed in all meals (pooled across diets). No covariates were entered. Both carbohydrate-to-fat blend index and fruit and vegetable scores are highly significant independent predictors (Supplemental Table 1), with the former and latter associated with high- and lower energy meals (kcals), respectively. Together, and in combination with meal type (breakfast, lunch, or dinner), across both diets, modeled meal energy intakes are highly correlated (*r* = 0.78, df = 1676, *P* < 0.001) with observed ad libitum energy intakes (Figure 7), with a mean absolute model error of 171.8 kcal, which is 22% of the mean meal size.

Next, to estimate the absolute impact on energy intake, for each diet, we took the average carbohydrate-to-fat ratio blend index and the average fruit and vegetable score and used our model to predict meal sizes. Respectively, for the ultraprocessed diet, this returned 689 kcal, 896 kcal, and 883 kcal for breakfast, lunch, and dinner. For the unprocessed diet, our model returned estimates of 584 kcal, 810 kcal, and 785 kcal, at breakfast, lunch, and dinner, respectively. Across 3 meals per day, this amounts to a 289.2 kcal difference, which means our 2 predictors account for 87.6% of the difference in daily nonbeverage energy intake (330 kcal) that was observed in the nonbeverage food intake data collected by Hall et al. [1].

### Micronutrient deleveraging

A potential benefit of selecting low-energy-dense meal components is that they are rich in micronutrients. With respect to components consumed, when %RDA values are summed, we see that the unprocessed diet delivered 36% more micronutrients than the ultraprocessed diet. Figure 6B shows how these micronutrients were distributed across components with different energy densities. Again, a clear pattern is observed—in the ultraprocessed diet, only 5% of the micronutrients were delivered by meal components <1.0 kcal/g. By contrast, in the unprocessed diet, meal components <1.0 kcal/g delivered 42% of the micronutrients consumed.

**FIGURE 8:**
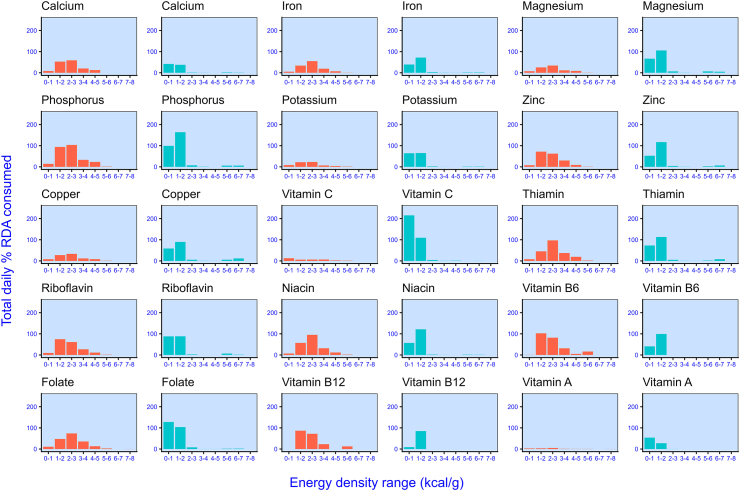
RDA (%) values for 15 micronutrients consumed in the ultraprocessed (orange) and the unprocessed (turquoise) diets, separately. Values were computed by totaling the % RDA consumed across the entire diet by all participants, and then dividing this amount by the diet duration (14 d) and the number of participants (*N* = 20). Any single component delivering >100% RDA was capped at 100%. RDA, recommended daily allowance.

To develop this further, we also explored these distributions for individual micronutrients. Figure 8 shows the extent to which daily RDA was met by components that differ in energy density. Values were computed by summing the %RDA consumed in each diet. Because amounts above 100% RDA are likely to be excreted, in any single meal, a component delivering >100% RDA was capped at 100%. Then, for each micronutrient, we derived an estimate of the extent to which the %RDA was met for a single participant on any given test day [total/(20 participants × 14 test days)]. In the unprocessed diet, in every case, lower energy-dense components (<2.0 kcal/g) delivered the majority of micronutrient intakes, and a large proportion was derived from components with an energy density <1.0 kcal/g (i.e., fruit and vegetables). By contrast, in the ultraprocessed diet, micronutrients were provided by relatively energy-rich components, some of which were micronutrient-fortified (e.g., breakfast cereals).

Together, these data show that in the unprocessed diet, selecting low-energy-dense, high-carbohydrate meal components was associated with meals of greater mass but lower energy. This pattern also appeared to ensure that micronutrient requirements were met. In turn, this assumes that micronutrient requirements could not have been met by consuming the more energy-dense components that were left uneaten at the end of each meal. To test this, we replotted Figure 8, this time showing the %RDA that remained in uneaten meal components. Consistent with our hypothesis, Supplemental Figure 2 shows that in the unprocessed diet, micronutrients were not generally available in more energy-rich components. Thus, to achieve adequate micronutrient intakes, it was necessary to consume low-energy-dense components. By contrast, in the ultraprocessed diet, micronutrients were colocated in more energy-rich components.

## Discussion

In this secondary analysis, we explored why consuming an unprocessed diet might limit or cap energy intake. As hypothesized, participants tended to combine components that delivered a meal-level blend of energy from carbohydrate and fat. However, less-equal blends were associated with the consumption of lower energy meals, and this was more common in the unprocessed diet. Also, as hypothesized, the lower energy density of the unprocessed meals was driven by the preferential selection of micronutrient-rich components—primarily fruits and vegetables. Prioritizing these foods over more energy-dense alternatives had the effect of “deleveraging” energy intake.

Across both diets, we observed a clear tendency for meals to have a higher carbohydrate-to-fat blend index than the components from which they were formed, and when a more equal blend is achieved, higher energy meals are consumed. In turn, this supports the idea of a process whereby meal components are selected and combined to promote energy intake by increasing the energy-to-satiety ratio of a meal [6]. This tendency was more pronounced with ultraprocessed meals, and it was associated with higher energy intake. So far as we know, this is a new mechanism by which ultraprocessed foods might encourage excess energy intake and deserves further study [6]. Although this strategic blending of components has not been previously documented, historical evidence suggests that humans have been doing this for a long time, with references to meals comprising sources of fat and carbohydrate dating back to the 18th [22], 15th [23], and 14th [24] centuries. On this basis, we contend that the general tendency to blend carbohydrate and fat in a meal is not unique to the modern food environment.

As previously noted, we suggest that this history of blending carbohydrate and fat might reflect an underlying functional advantage—maximizing energy intake [6]. However, the modern food environment may exploit this tendency, leading to overconsumption, obesity, and its associated comorbidities. Future research might examine these relationships in other datasets and in randomized controlled trials that systematically manipulate the blend of individual meal components to assess the effect on component selection, meal composition, and energy intake.

These data also show that food choice is driven by factors beyond macronutrients or their strategic combination. Given the widespread assumption that humans tend to prefer energy-rich foods [2], it is notable that substantial amounts of pasta, cream, olive oil, steak, spaghetti, hash browns, potatoes, and couscous were left uneaten in the unprocessed diet. Furthermore, large portions of vegetables such as spinach, broccoli, and green beans were eaten, many in portions exceeding 500 g. Indeed, these low-energy-dense components comprised 52% of the total mass of unprocessed components consumed.

Previous laboratory studies show a linear relationship between food energy density and both acute [25,26] and daily [27] energy intake in children and adults. Consequently, when food energy density is manipulated, it has little impact on the mass of food consumed [16], suggesting insensitivity to food energy density. From this, one would expect that the mass of ultraprocessed and unprocessed meals would be similar. Instead, the unprocessed meals were much larger (57%).

Related to energy density are observations that food texture influences eating rate [28] and eating faster is associated with larger meals [29]. Hall et al. [1] reported that the ultraprocessed meals were consumed faster (48 kcal/min, 37 g/min) than unprocessed meals (31 kcal/min, 30 g/min) [1,30]. Accordingly, one hypothesis is that Hall et al.’s [1] ultraprocessed meals were higher in calories because they were softer and less fibrous [30]. If unprocessed meals require more oral processing and thus are eaten more slowly, and if slower eating leads to smaller meals (by mass), then it follows that unprocessed meals should have been smaller (by mass) than ultraprocessed meals. Again, our analysis shows the converse—they were significantly larger (57%).

Energy density and eating rate may still influence energy intake. However, they cannot explain the large mass of low-energy-dense unprocessed components consumed, or their role in limiting energy intake. As we have seen, this was governed mainly by the selection of fruit and vegetables, despite the availability of more energy-dense components. Thus, the lower energy density of the unprocessed diet should be viewed as a consequence of choice rather than a driver of reduced intake.

Regarding micronutrients, 2 characteristics of the diets merit consideration. First, consistent with previous reports [31,32], the unprocessed meals delivered more micronutrients overall. However, contrary to the widely held belief that ultraprocessed foods deliver “empty calories” [33], Figure 8 shows that both diets probably delivered sufficient micronutrients to meet most requirements. Second, the distribution of these nutrients differed markedly between diets. In the unprocessed diet, a substantial proportion was delivered by foods with a low or very low-energy density, whereas the ultraprocessed diet featured meal components rich in both calories and micronutrients. For example, baby carrots and spinach delivered the largest amounts of vitamin A in the unprocessed meals. In the ultraprocessed meals, by comparison, the components richest in vitamin A were French toaster sticks and pancakes.

Thus, in the ultraprocessed diet, calories and micronutrients were colocated within the same components, so requirements for both were met by consuming the same foods. By contrast, in the unprocessed diet, low-energy-dense components were needed to meet micronutrient requirements, which restricted the energy density of the unprocessed meals and increased their volume. It is plausible that without consuming these very low-energy-dense (<1.0 kcal/g) foods, diet quality would have been compromised and, over time, micronutrient insufficiencies would have occurred. Thus, an unprocessed diet may create a previously unrecognized tension between maximizing calories and meeting nutritional needs. Accordingly, it is possible that the selection of low-energy-dense, micronutrient-rich components had a deleterious effect on energy intake. Similar to “protein leveraging” [34], “micronutrient deleveraging” might occur because the opportunity cost of meeting essential nutritional requirements is the need to forego calories (see [35] for a related discussion). Unlike protein leveraging, however, this compromise results in reduced energy intake.

In turn, ultraprocessed foods may promote energy intake because fortification resolves the tension between meeting calorie and micronutrient needs—a constraint inherent in whole-food diets. A parallel exists in pig farming: historically, pigs were fed soy and corn, supplemented with pasture to prevent deficiencies. From the late 1940s, micronutrient fortification allowed farmers to eliminate the need for feed rations to be supplemented with micronutrient-rich low-energy pasture or forages [36]. This improved energy intake and growth rates [37] by increasing the total energy density of the diet [38].

This work offers a new interpretation of Hall et al.'s [1] data, but like others, it has limitations. Regarding the tendency to select low-energy-dense micronutrient-containing components, these findings build on evidence in other species [12,39] but reveal nothing directly about mechanisms. Our analysis also assumes that an optimal energy-to-satiety ratio is achieved when meal components combine to yield equal energy from carbohydrate and fat. Although such blending has been observed in rodents [40], there is no a priori reason to assume this is optimal—an assumption rooted in the idea that energy from carbohydrate and fat is equally satiating [6]. Further research is needed to clarify the optimal ratio and whether subtle shifts in this ratio can arise from individual differences or nutritional context.

Our analysis also suggests the nutritional nature of unprocessed and ultraprocessed foods—and our relationship to them—tells a more nuanced story. Rather than simply being attracted to energy-dense foods, we suspect humans discriminate based on a process that increases energy intake by optimizing energy-to-satiety ratio, which is partly achieved by blending sources of carbohydrate and fat. With an unprocessed diet, this is more challenging because meal components are less “preblended,” and, in addition, our nutritional intelligence may promote the inclusion of low-energy-dense fruit and vegetables.

Finally, we remind readers that this work represents a reanalysis of data collected to address a different scientific question. Although our observations align with our hypotheses, further research is needed to confirm them definitively. Nevertheless, given widespread concern over the modern food environment and its impact on obesity, we believe these ideas warrant further investigation. In this regard, researchers should extend beyond NOVA to investigate other processing frameworks [41] while exploring how an unprocessed diet leads to healthier, lower energy meals.

## Author contributions

The authors’ responsibilities were as follows – KDH, ABC: designed and conducted the original research on which this work is based; JMB, ANF, PJR, ABC, MS: designed the post-hoc reanalysis strategy, and JMB analyzed the data; all authors: wrote the paper; and JMB: had primary responsibility for final content; and all authors: read and approved the final manuscript.

## Data availability

Data described in the manuscript will be made available upon request, pending application and approval.

## Funding

JMB and ANF are partly supported by the National Institute for Health and Care Research Bristol Biomedical Research Centre and the National Institute for Health and Care Research Applied Research Collaboration West (NIHR ARC West). ABC is supported by the Intramural Program of the National Institute of Diabetes & Diabetes & Kidney Diseases at the National Institutes of Health, Bethesda, Maryland.

## Conflict of interest

Jeffrey Brunstrom reports financial support was provided by NIHR Bristol Biomedical Research Centre. Kevin Hall reports a relationship with AstraZeneca PLC that includes: consulting or advisory. Kevin Hall reports a relationship with MacrosFirst that includes: consulting or advisory. Kevin Hall reports a relationship with Weight Watchers that includes: consulting or advisory. Co-author Mark Schatzker receives royalties as the author of books related to human dietary behavior.
