# Supplementary material for: Consuming an unprocessed diet reduces energy intake: a post-hoc analysis of a randomized controlled trial reveals a role for human nutritional intelligence
Source: Am J Clin Nutr. 2025 Dec 29;123(3):101183. doi: 10.1016/j.ajcnut.2025.101183 (PMC12975374; doi:10.1016/j.ajcnut.2025.101183)

**Supplementary Materials**

Consuming an unprocessed diet reduces energy intake: A post-hoc analysis of a randomized control trial reveals a role for human nutritional intelligence

Jeffrey M. Brunstrom

**
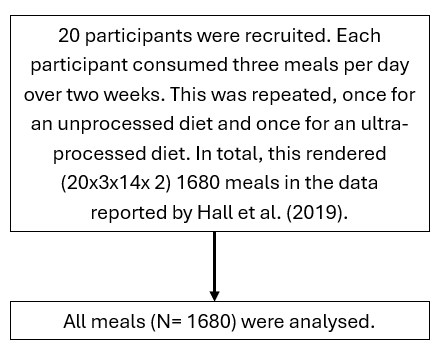
**

**Supplementary Figure 1:** Flowchart illustrating the selection of data included for analysis.


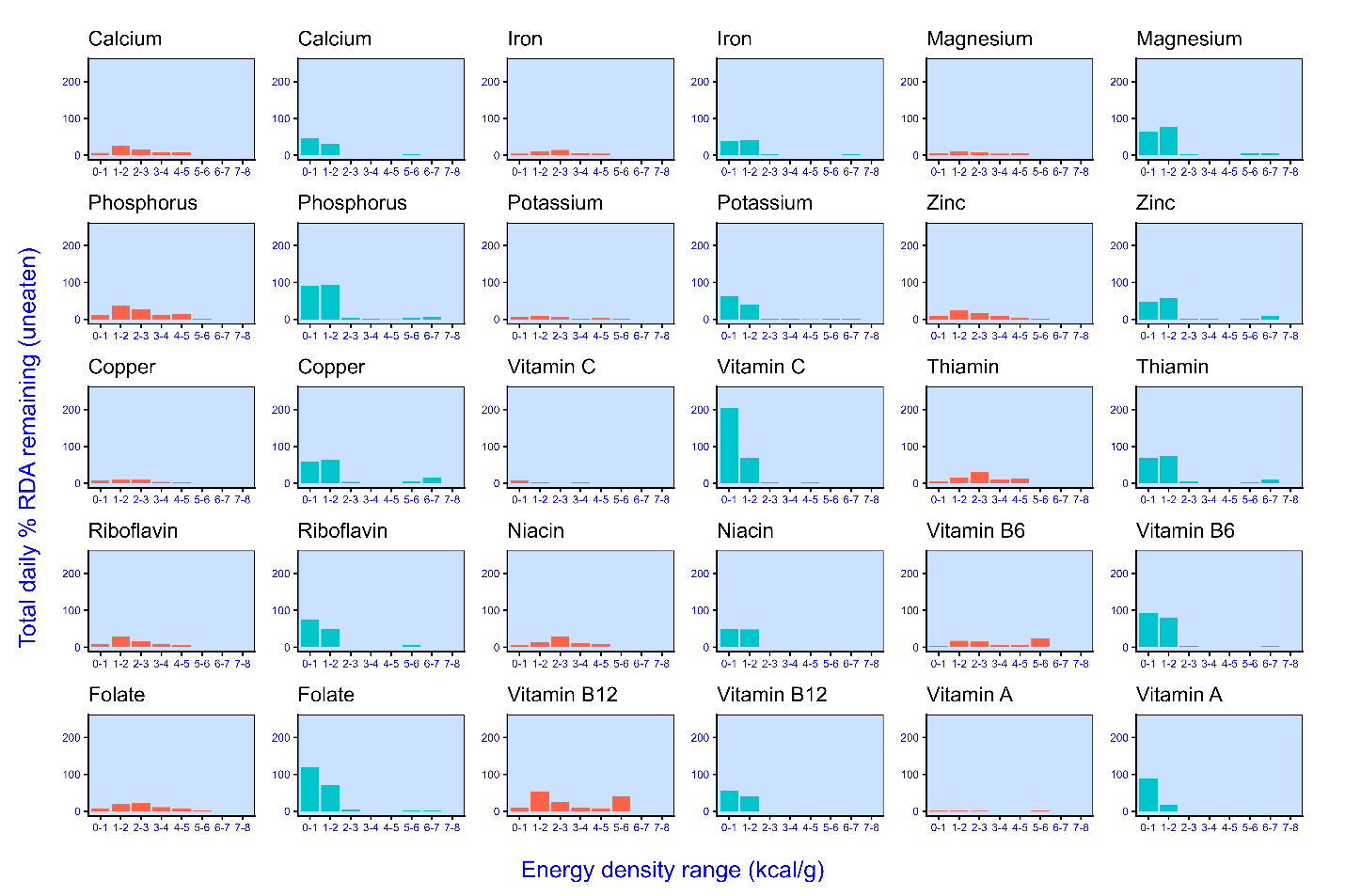


**Supplementary Figure 2**: RDA (%) values for 15 micronutrients remaining (uneaten) in the ultraprocessed (orange) and unprocessed (turquoise) diets, separately. Values were computed by totaling the % RDA consumed across the entire diet by all participants, and then dividing this amount by the diet duration (14 days) and the number of participants (*N* = 20). Any single component delivering more than 100% RDA was capped at 100%. RDA, recommended daily allowance.

**Supplementary Table 1**

Linear mixed-effect model of *ad libitum* meal energy intake.

|  | Standardized *β* | Standard error | *P* value | *T* value | *95% CI* |
| --- | --- | --- | --- | --- | --- |
| Both diets (1680 meals; *N* = 20) | | | | |  |
| Carb-fat ratio | 52.93 | 5.65 | <0.0001 | 9.36 | 41.9 to 64.0 |
| Fruit & veg score | -80.61 | 5.64 | <0.0001 | -14.28 | -91.7 to -69.5 |

**Q-Q plots for data in Figure 3A**


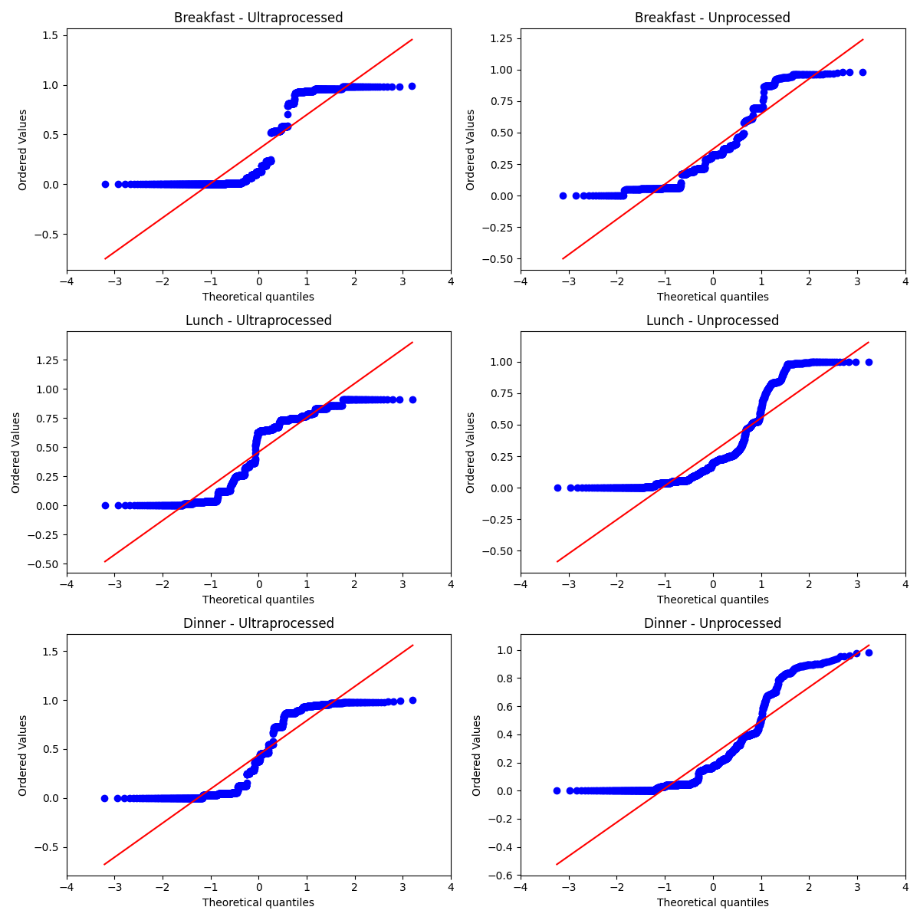


**Q-Q plots for data in Figure 3B**


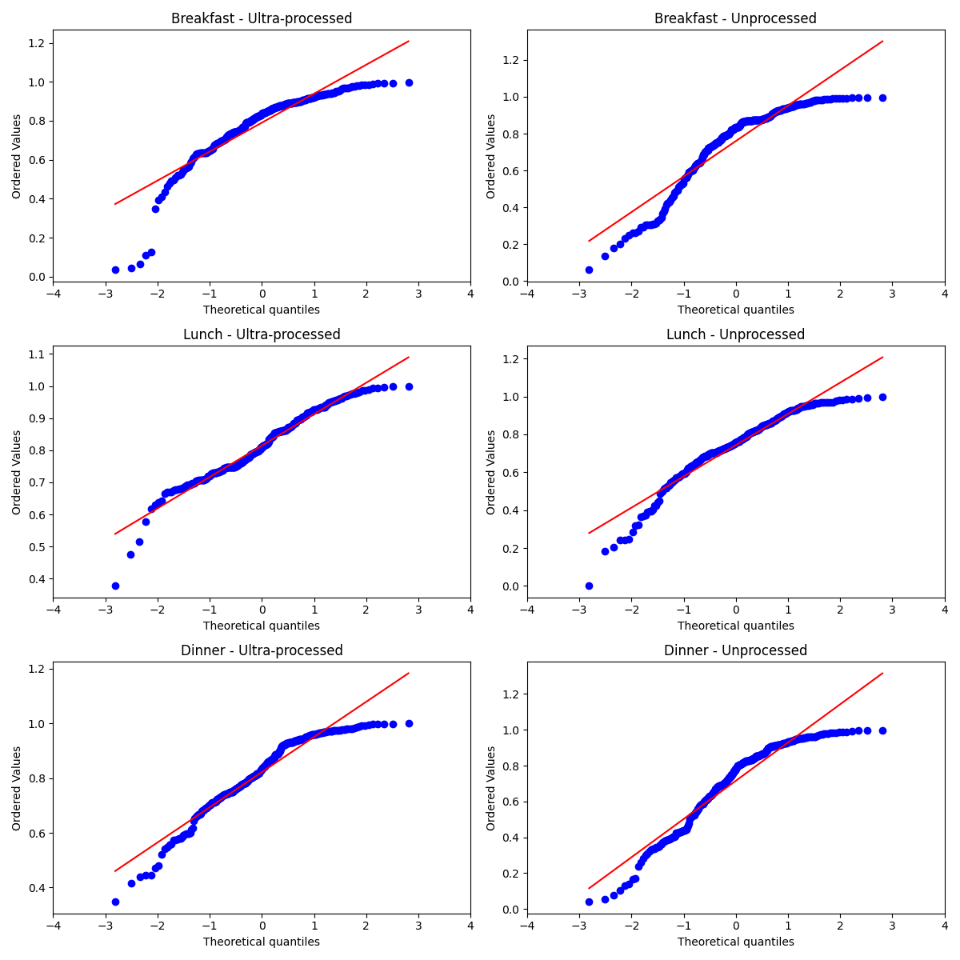


**Q-Q plots for data in Figures 5A and 5B**


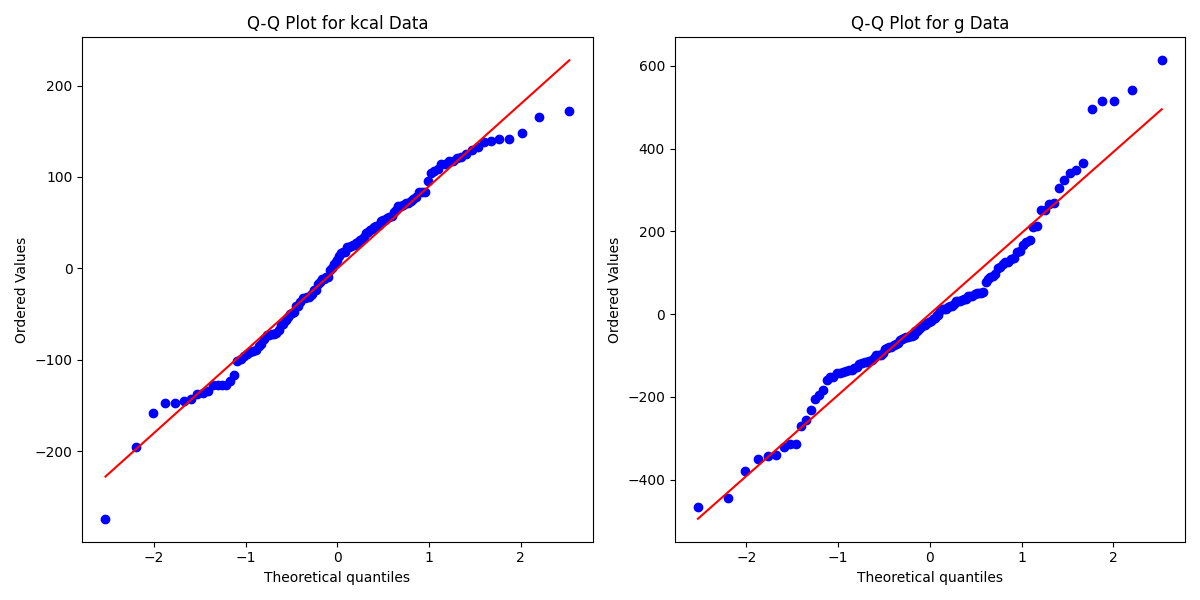


**Q-Q plot for data in Figure 7**


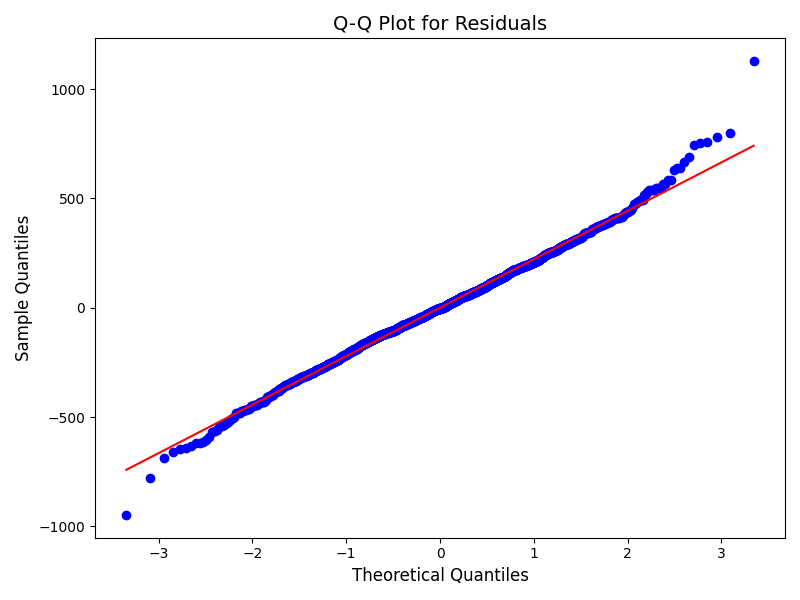

Supplement: multimedia component 1 [file mmc1.docx]
